# Intracranial manifestations of rhinocerebral mucormycosis: a pictorial essay

**DOI:** 10.1186/s43055-022-00765-5

**Published:** 2022-04-12

**Authors:** Alka Agrawal, Prem S. Tripathi, Prachi Shukla, Prakhar Nigam, Pramita Kheti

**Affiliations:** Department of Radiodiagnosis, M.G.M. Medical College & M.Y. Hospital, CRP Line, Indore, Madhya Pradesh 452001 India

**Keywords:** Mucormycosis, Magnetic resonance imaging, COVID-19

## Abstract

Rhinocerebral mucormycosis has emerged as a common coinfection in coronavirus disease 2019 (COVID-19) patients during the convalescence period. Frequent spread of disease from sinonasal mucosa to bone, neck spaces, orbit, and brain occurs along the perivascular/perineural routes or through direct invasion. Brain involvement represents severe manifestation and is often associated with poor functional outcomes and high mortality rates. Magnetic resonance imaging (MRI) is the modality of choice for the intracranial assessment of disease severity in mucormycosis. Early and accurate identification of intracranial extension is imperative to improve survival rates. With this pictorial essay, we aim to familiarize the readers with the cross-sectional imaging features of intracranial complications of mucormycosis. The radiological details in this essay should serve as a broad checklist for radiologists and clinicians while dealing with this fulminant infection.

## Background

Rhinocerebral mucormycosis (RCM) is caused by invasive fungi belonging to the order Mucorales [1]. The genera Rhizopus, Mucor, and Lichtheimia of this order account for 70% to 80% of reported mucormycosis cases [2]. The primary route of infection is by inhalation of sporangiospores, the asexual spores of Mucorales, leading to invasive sinopulmonary infection in susceptible individuals [3]. Individuals susceptible to infection include those with uncontrolled diabetes mellitus, hematological malignancies, solid organ transplantation, and immunocompromised state [4]. Unlike the situation in developed countries, where malignancy has emerged as the major risk factor for mucormycosis, uncontrolled diabetes mellitus has been the dominant driver of disease in developing nations [5–7].

Coronavirus disease 2019 (COVID-19) has emerged as another major risk factor for RCM since the start of the pandemic in 2020. Severe acute respiratory syndrome coronavirus-2 (SARS-CoV-2) infection impairs innate and adaptive immunity, worsens hyperglycemia in diabetics, increases serum free iron levels, and induces endothelial receptors for entry of the Mucorales [8–11]. All these along with the rampant use of steroids for the treatment of COVID-19 create a perfect environment for fungal growth and invasion.

The disease usually begins with sinonasal symptoms—headache, fever, nasal congestion and rhinorrhea, epistaxis, nasal hypoesthesia, facial pain, and numbness. Orbital involvement through direct spread or vascular routes causes orbital or periorbital pain, amaurosis, diplopia, blurring of vision, and ophthalmoplegia [12]. CNS penetration of sinonasal disease occurs by direct extension from paranasal sinuses or through blood vessels or nerves [3]. Convulsions, dizziness, altered mental status, and hemiparesis signal brain involvement [12].

A direct microscopy, histopathology, and fungal culture of surgical specimens form the cornerstones in the diagnosis of mucormycosis. Non-septated, branching hyphae of Mucorales can be demonstrated under direct microscopy [13]. All Mucorales species grow well on Sabouraud dextrose agar at a temperature of 25–30 °C [13]. Neutrophilic or granulomatous inflammation, necrosis, and angioinvasion are predominant findings on pathological specimens [13]. Histopathological specimen from the brain to confirm central nervous system (CNS) involvement seems pointless as it may lead to neurological deficits. Therefore, the diagnosis of CNS mucormycosis is frequently made indirectly by identifying the pathogen in the sinuses [3].

The diagnosis of intracranial presence and extent of the disease relies mainly upon imaging in confirmed cases of sinonasal mucormycosis. Early radiological diagnosis provides the opportunity for prompt initiation of medical treatment and timely surgical debridement. Magnetic resonance imaging (MRI) is the modality of choice because of its brilliant soft tissue resolution which aids in the identification of disease extension with high precision. The pictorial essay provides an insight into the possible MRI appearances of cerebral mucormycosis, general characteristics, and key identification features.

## Main text

An appropriate imaging protocol is a prerequisite for the optimal reporting of radiological data. In suspected cases of sinonasal mucormycosis, coronal T1-weighted, T2-weighted fat-saturated, and T1 fat-saturated contrast-enhanced images from nose tip to brainstem [field of view (FOV) 180 mm]; axial T1-weighted, T2-weighted fat-saturated, and T1 fat-saturated contrast-enhanced from top of hyoid to frontal sinuses (FOV 180 mm) should be performed. Additional oblique sagittal T2 fat-saturated and contrast-enhanced imaging of orbits should be performed in patients with orbital lesions. Imaging of the brain with axial, sagittal, and coronal T1-weighted, T2-weighted, fluid-attenuated inverse recovery (FLAIR) sequence, and T1 fat-saturated contrast-enhanced imaging should be added in for intracranial extension. Fat-saturated contrast-enhanced images help in the evaluation of periantral, orbital, and intracranial lesions along with identification of cavernous sinus thrombosis/thrombophlebitis, internal carotid arteritis, and thrombosis. Fat-saturated fast spin-echo (FSE)/short tau inverse recovery (STIR) sequence depicts marrow signal changes with the involvement of skull base. Diffusion-weighted imaging for assessment of cranial nerves for infarction, cerebritis, intracerebral abscess, and cerebral infarcts is advisable. 3D post-contrast T1-weighted images help in establishing perineural spread through the abnormal sheet of tissue along the nerve with the enhancement of nerve sheath and vascular invasion through wall enhancement and/or luminal thrombosis. Time-of-flight (TOF) magnetic resonance angiogram (MRA) should be performed in vascular thrombosis to assess the degree and extent of involvement.

The involvement of extrasinus tissue in rhinocerebral mucormycosis occurs secondary to the sinonasal disease. The identification of fungal etiology and early signs of disease extension outside the paranasal sinuses aids in the diagnosis of invasive fungal sinusitis. On computed tomography (CT), hyperdense lesions in paranasal sinuses, nodular mucosal thickening, and absence of air-fluid level suggest fungal etiology [14]. CT allows a fair assessment of premaxillary and retroantral fat, one of the earliest and most common sites for fungal invasion which can occur without any obvious bony rarefaction or dehiscence of maxillary sinus walls [14, 15]. Bone involvement in the form of erosion, expansion, or thinning is reliable indicators for invasiveness and can readily be demonstrated on CT [15, 16]. The assessment of fungal spread to neck spaces, orbit, and brain on CT is difficult and is assisted by contrast-enhanced imaging.

**Fig. 1:**
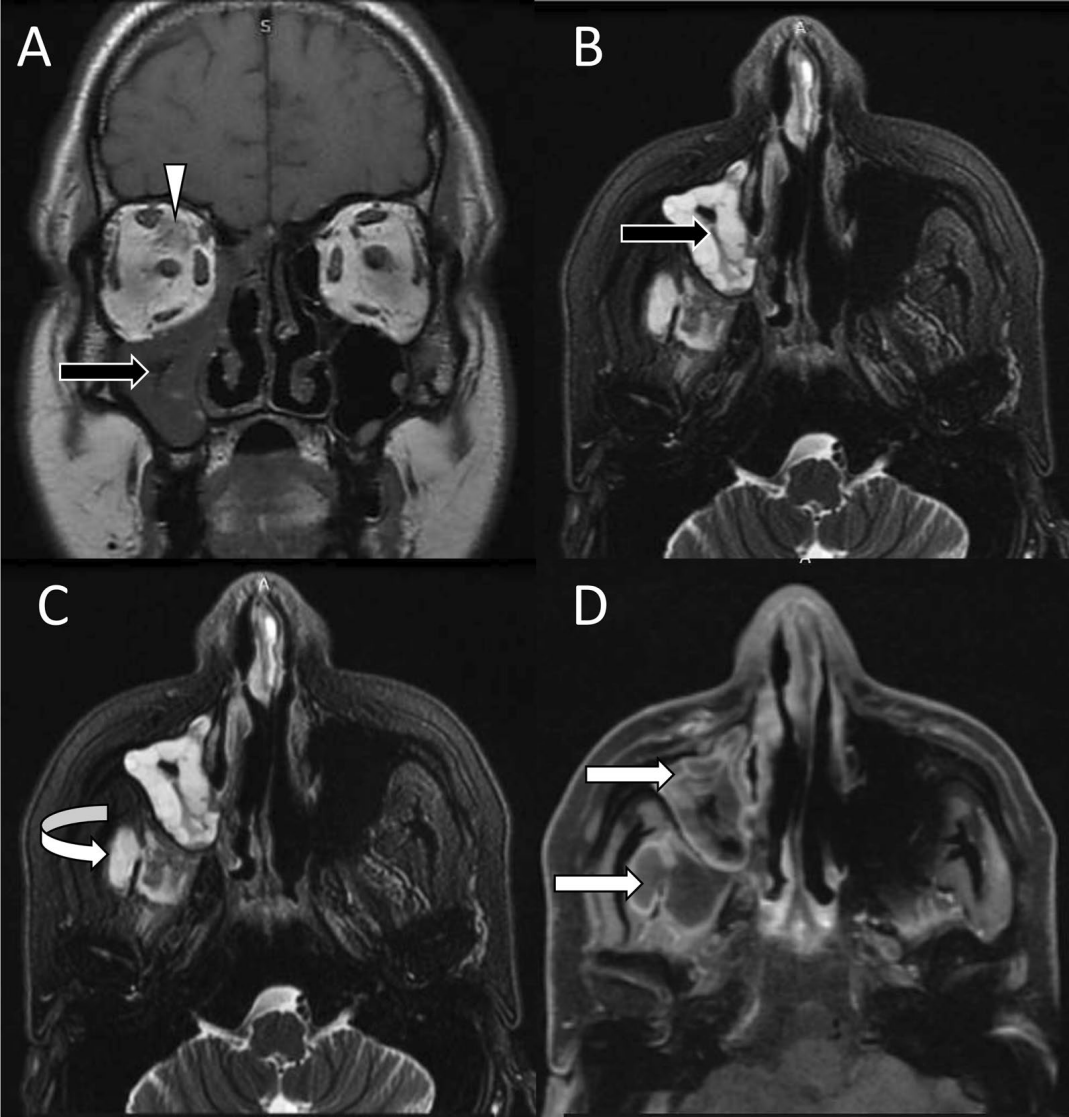
Sinus mucormycosis with extension to orbit and neck. Coronal T1-weighted (**A**) and axial T2-weighted fat-saturated (**B**, **C**) images show T1 hypointense and T2 hyperintense soft tissue (black arrows) in the right maxillary sinus extending posteriorly into retromaxillary neck space (curved white arrow). Contrast-enhanced axial T1-weighted fat-saturated image shows peripheral enhancement with central non-enhancing area (straight white arrows). Note the intraconal fat stranding in the image (**A**) suggesting orbital involvement (white arrowhead)

On MRI, the fungal elements appear characteristically hypointense on T2-weighted images. However, intermediate or high signals on T2-weighted images are not uncommon [17]. The ‘black turbinate sign’ which is lack of enhancement of mucosa due to fungal invasion and devitalization has been classically described in invasive fungal sinusitis [18]. The black turbinate is an early sign of invasive fungal sinusitis [18, 19]. It should be differentiated with benign black turbinate seen in normal individuals. Involvement of the posterior portions of the inferior and middle turbinates, well-defined margins, hyperintense T2 signals, thin peripheral enhancement, progressive enhancement on dynamic contrast-enhanced scans, and T2 hyperintensity suggest benign black turbinate [18, 19]. MRI due to its excellent soft tissue resolution provides a superior assessment of extrasinus fungal spread in comparison with CT. The presence of abnormal soft tissue in nasolacrimal ducts, extraconal fat, and pterygopalatine fossa indicates imminent orbital and cerebral mucormycosis [16]. Post-contrast images are indispensable for the assessment of sinus and extrasinus fungal lesions (Fig. 1). Contrast-enhanced images may also uncover the non-enhancing devitalized tissues, abnormal enhancement of neck and orbital muscles, perineural and vascular lesions.

Intracranial spread of fungal infection suggests the fulminant course of the disease and is usually associated with poor prognosis [3]. Therefore, accurate identification of intracranial lesions is necessary to improve the disease outcome. A myriad of lesions is possible with intracranial involvement of mucormycosis which has been discussed in detail in this essay.

### Cerebritis

**Fig. 2:**
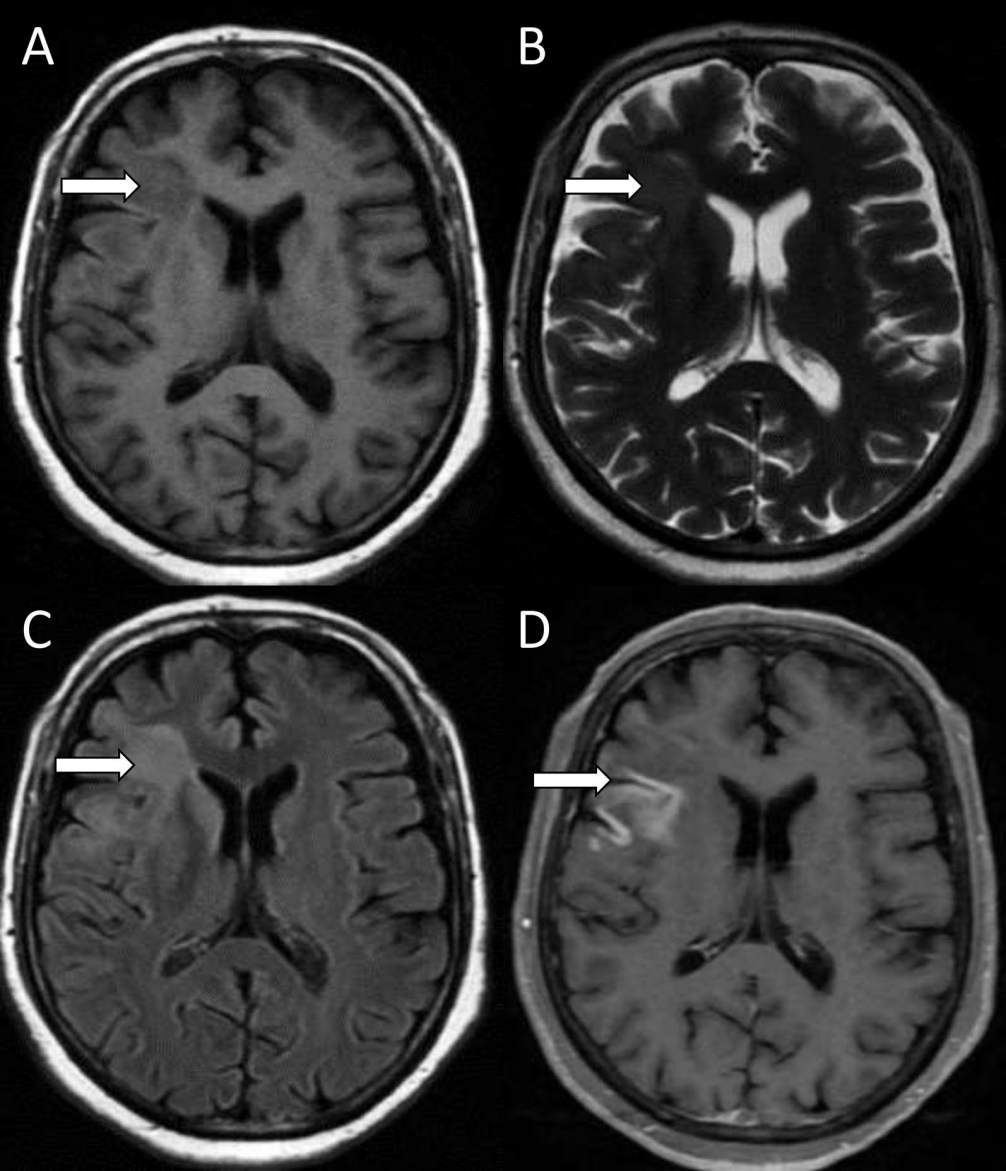
Rhinocerebral mucormycosis with cerebritis. Axial T1-weighted image (**A**) shows an isointense lesion in the right frontal lobe (white arrow). It appears hyperintense on axial T2-weighted (**B**) and FLAIR (**C**) images and shows enhancement on contrast-enhanced axial T1-weighted fat-saturated image (**D**)

**Fig. 3:**
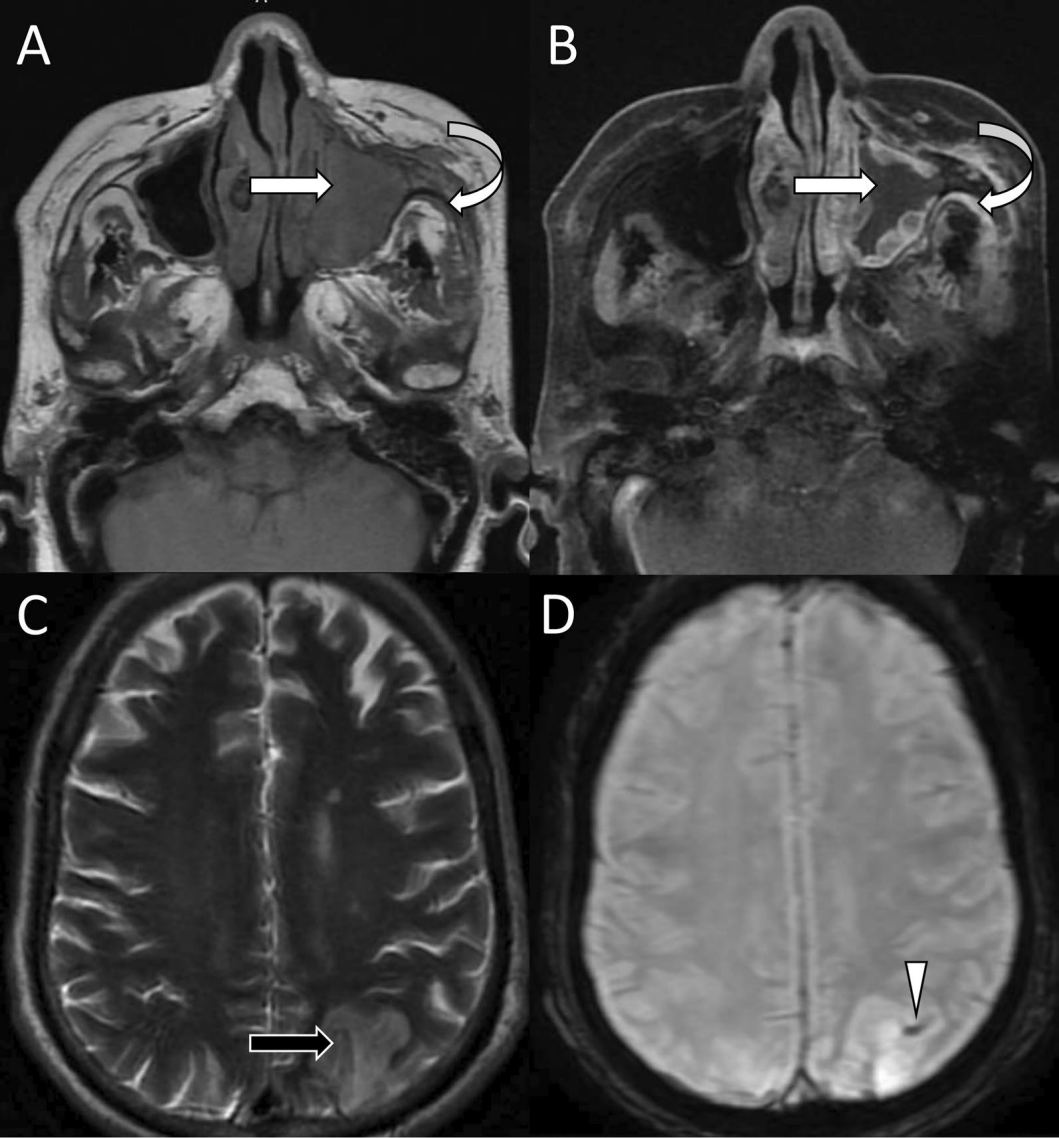
Rhinocerebral mucormycosis with late cerebritis. Axial T1-weighted image (**A**) shows soft tissue in the left maxillary sinus (straight white arrow) with extension into left retroantral fat (curved white arrow). Contrast-enhanced axial T1-weighted fat-saturated image (**B**) shows peripheral enhancement of the maxillary lesion with intense enhancement of retroantral fat. Axial T2-weighted (**C**) and susceptibility-weighted (**D**) images show a heterogeneous hyperintense lesion in the left parietal lobe (black arrow) which shows peripheral foci of blooming (arrowhead)

**Fig. 4:**
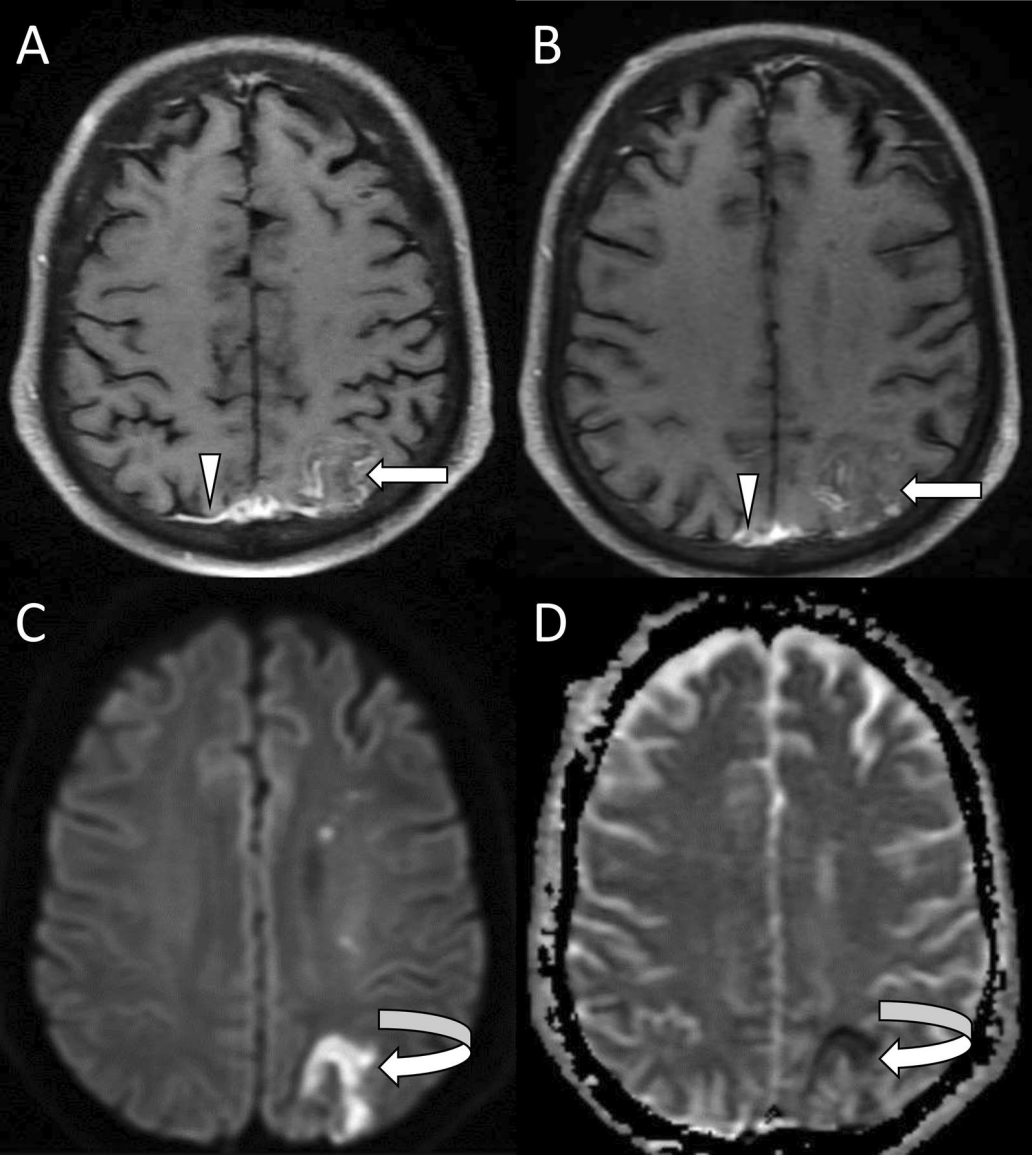
Rhinocerebral mucormycosis with late cerebritis. On contrast-enhanced axial T1-weighted fat-saturated images (**A**, **B**), the lesion shows mild incomplete peripheral enhancement (straight white arrows) with enhancement of the adjacent dura (arrowhead). Diffusion-weighted and apparent diffusion coefficient (ADC) images (**C**, **D**) show peripheral areas of restricted diffusion (curved white arrows)

The anterior and middle cranial fossa are the common sites of parenchymal involvement. Fungus gain access by eroding cribriform plate of ethmoid or along fibers of olfactory nerve [20, 21]. The infection may also gain access to frontoparietal brain matter through blood vessels. Early cerebritis appears as T1 iso/hypointense, T2/FLAIR hyperintense lesion with no post-contrast enhancement, mainly in the cortical and subcortical areas. The lesion may show patchy areas of diffusion restriction on diffusion-weighted imaging (DWI). Mild or incomplete peripheral enhancement may be seen in later stages of cerebritis before evolving into a fungal abscess (Figs. 2, 3, 4) [22].

### Abscess

**Fig. 5:**
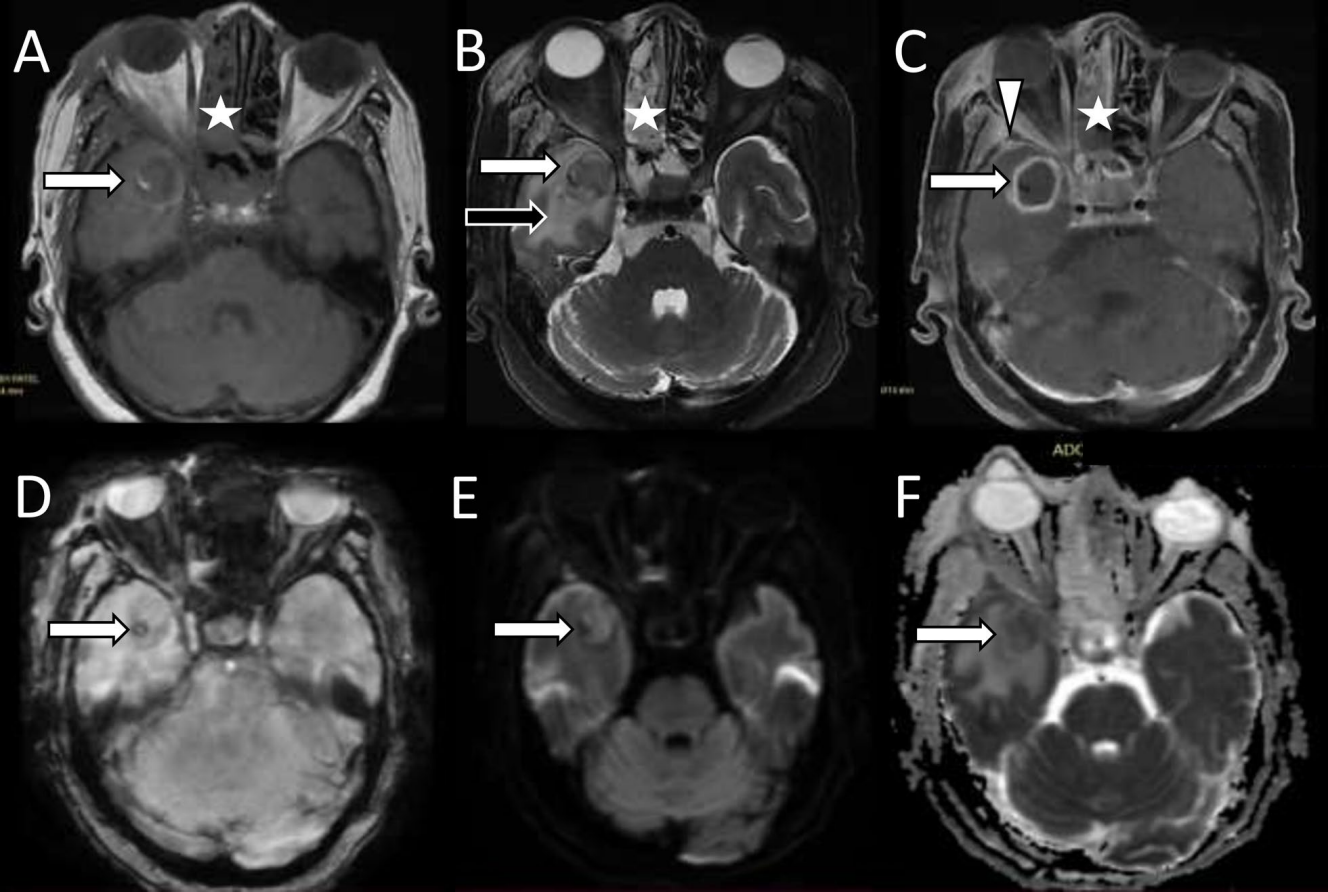
Fungal cerebral abscess. Axial T1-weighted image (**A**) shows a well-defined isointense lesion in the right temporal lobe (white arrow). Axial T2-weighted image (**B**) shows low signal (white arrow) with surrounding edema (black arrow). Contrast-enhanced axial T1-weighted fat-saturated image (**C**) shows ring enhancement of the lesion (white arrow) with associated meningeal enhancement (arrowhead). Susceptibility-weighted image (**D**) shows foci of blooming (white arrow) inside the lesion (likely fungal elements). Diffusion-weighted and apparent diffusion coefficient (ADC) images (**E**, **F**) show diffusion restriction with low ADC signal (white arrows). Note the soft tissue in right ethmoid sinuses suggesting their involvement (white stars in images **A**–**C**)

Intracerebral abscesses are commonly seen in the basifrontal region or anteromedial part of the temporal lobe [14]. The lesion appears T1 iso/hypointense, T2/FLAIR hyperintense with hypointense wall which shows ring enhancement on contrast administration (Fig. 5) [23]. At times fungal abscesses may show heterogeneous, incomplete annular, or no enhancement at all in severely immunocompromised patients with poor inflammatory response [24]. The abscess shows variable perilesional edema and mass effect. Diffusion restriction of the abscess wall and intracavitary projections is a characteristic finding on DWI. However, uniform restriction of the fungal core may occur and can be attributed to the presence of inflammatory cells and necrosis in the late capsular stage [25]. Magnetic resonance spectroscopy (MRS) demonstrates lipids (1.2–1.3 ppm), lactate (1.3 ppm), alanine (1.5 ppm), acetate (1.9 ppm), succinate (2.4 ppm), and choline (3.2 ppm) components within the fungal abscess. The presence of trehalose in the wall is considered a distinctive feature of fungal abscess [26, 27]. Susceptibility-weighted imaging (SWI) may show hypointense foci of hemosiderin deposits along the periphery or within the lesion [23].

### Cavernous sinus thrombosis

**Fig. 6:**
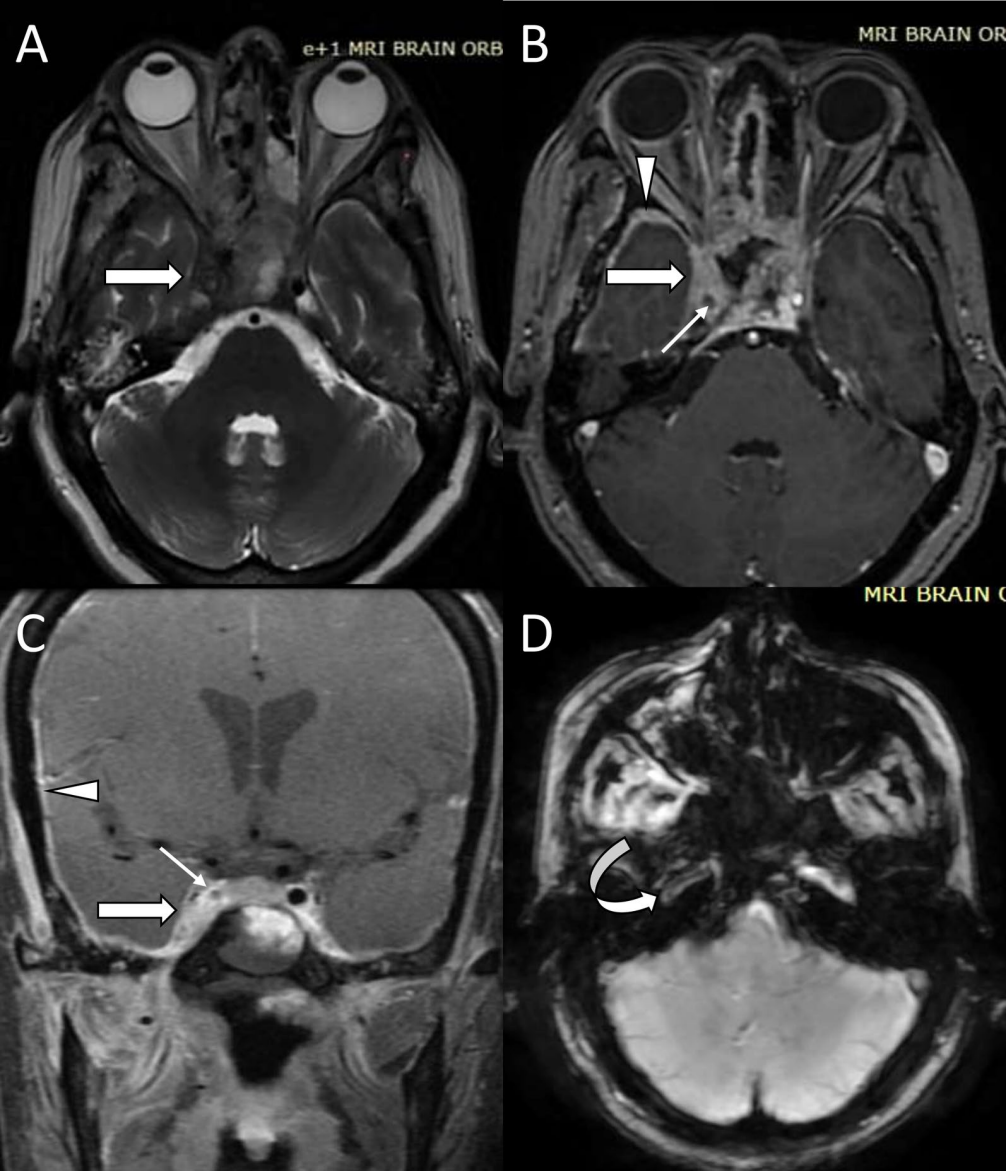
Right cavernous sinus (CS) and internal carotid artery (ICA) thrombosis. Axial T2-weighted (**A**), contrast-enhanced axial (**B**), and coronal (**C**) T1-weighted fat-saturated images show the bulging lateral border of the right cavernous sinus (white large arrows) with loss of normal flow void of the right internal carotid artery (white small arrows). The right CS shows filling defects on contrast-enhanced images indicating its thrombosis. Note the thickened and enhancing dura (white arrowheads) adjacent to the right temporal lobe. Left intracranial ICA shows normal flow void. Susceptibility-weighted image (**D**) shows blooming in the petrocavernous part of the right ICA suggestive of the presence of thrombus

Infection of ethmoid sinuses carries a high risk of cavernous sinus thrombosis because the valveless emissary veins draining this sinus allow fungal invasion of periorbital tissue, the orbital apex, and the cavernous sinus [28, 29]. Direct spread of disease with the involvement of orbital apex and sphenoid sinus is another possibility [28]. The lateral wall of the cavernous sinus normally appears straight or concave on axial and coronal images. In coronal contrast-enhanced images, the nerve traversing the wall and lumen of the cavernous sinus and the cavernous part of the internal carotid artery can be seen as non-enhancing foci and flow void, respectively. The presence of any other low signals is abnormal. Features suggestive of cavernous sinus thrombosis include bulge or convexity of the lateral wall, abnormal signal intensity on T1/T2-weighted images, and presence of filling defects on contrast administration (Fig. 6) [28, 30]. There may be associated superior ophthalmic vein occlusion, which can be identified as a dilated cord-like structure superior to the optic nerve crossing from the medial to the lateral side [21]. Thrombosed vein shows loss of normal flow void on unenhanced image and filling defects on post-contrast images [21]. The presence of abnormal enhancement of dura adjacent to sinus suggests pachymeningitis and soft tissue lesions in the paracavernous region denoted the lateral spread of disease to adjacent brain parenchyma.

### Internal carotid artery thrombosis

Partial or complete encasement of the cavernous part of the internal carotid artery by the soft tissue or thrombus present in the cavernous sinus may lead to luminal narrowing. There is a high propensity for arterial invasion by the Mucorales due to its ability to bind to the endothelium through specific receptors and invade the internal elastic lamina causing intimal damage and thrombosis [31, 32]. The vessel wall invasion can cause vasculitis without any visible thrombus which appears as mural thickening and vessel wall enhancement on post-contrast images. Further extension into the lumen causes thrombosis. Loss of normal flow void on T1/T2-weighted images and intraluminal hypointensity on SWI suggests luminal occlusion by a thrombus (Fig. 6). The carotid artery occlusion is one the most common causes of mucormycosis-associated cerebral ischemia and infarcts. It should be followed by an angiogram for better assessment of the length of the segment involvement [33].

### Cerebral infarcts and septic emboli

**Fig. 7:**
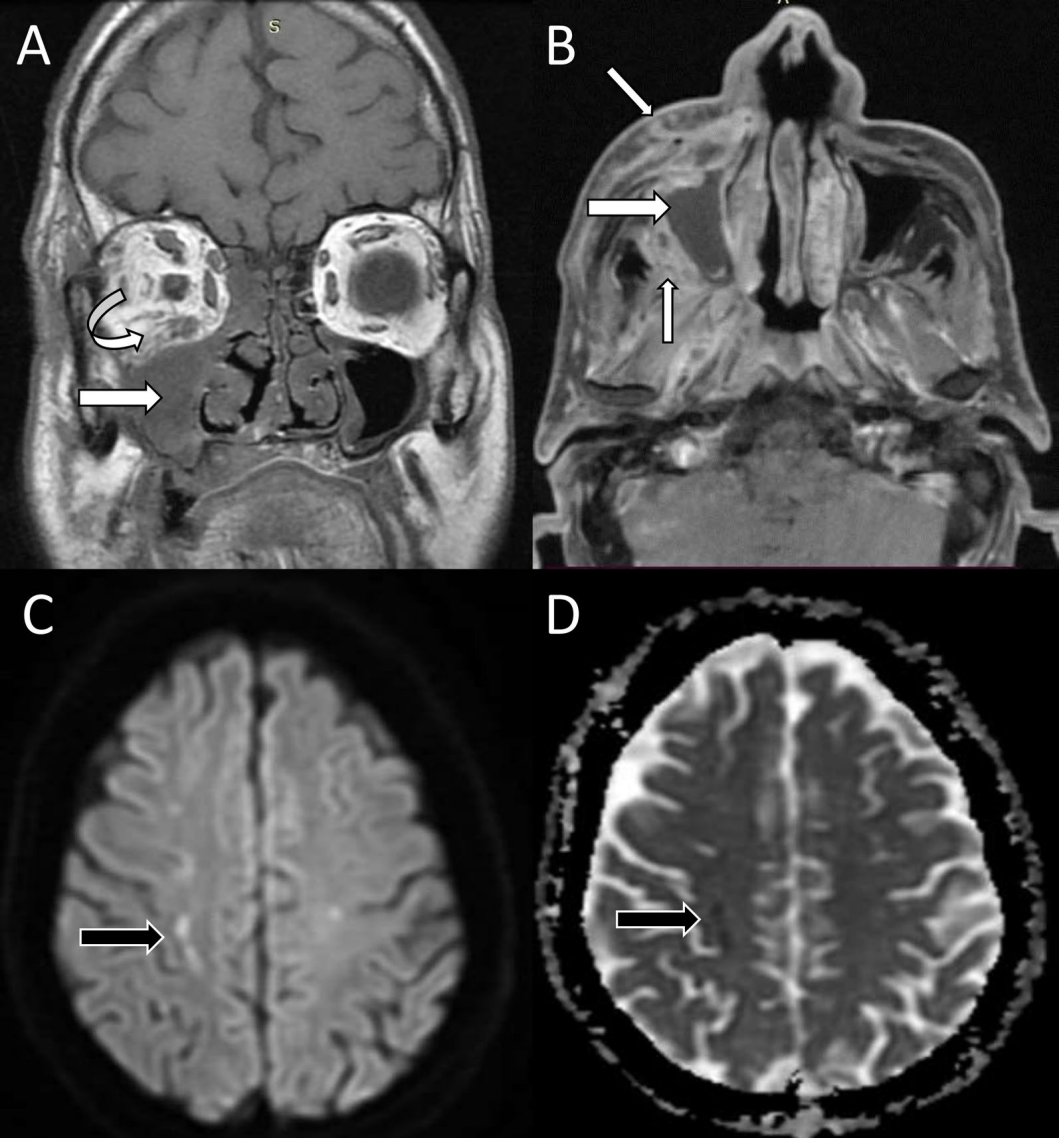
Rhinocerebral mucormycosis-associated cerebral infarct. Coronal T1-weighted image (**A**) shows soft tissue in the right nasal cavity and maxillary sinus (white arrow) with intraorbital fat stranding (curved arrow). Contrast-enhanced axial T1-weighted fat-saturated image (**B**) shows non-enhancing maxillary sinus lesion (large white arrow) with the heterogeneous enhancement of the periantral soft tissues (small white arrows). Diffusion-weighted and apparent diffusion coefficient (ADC) images (**C**, **D**) show foci of diffusion restriction with corresponding low ADC signal (black arrows) in the right centrum semiovale in the watershed territory

**Fig. 8:**
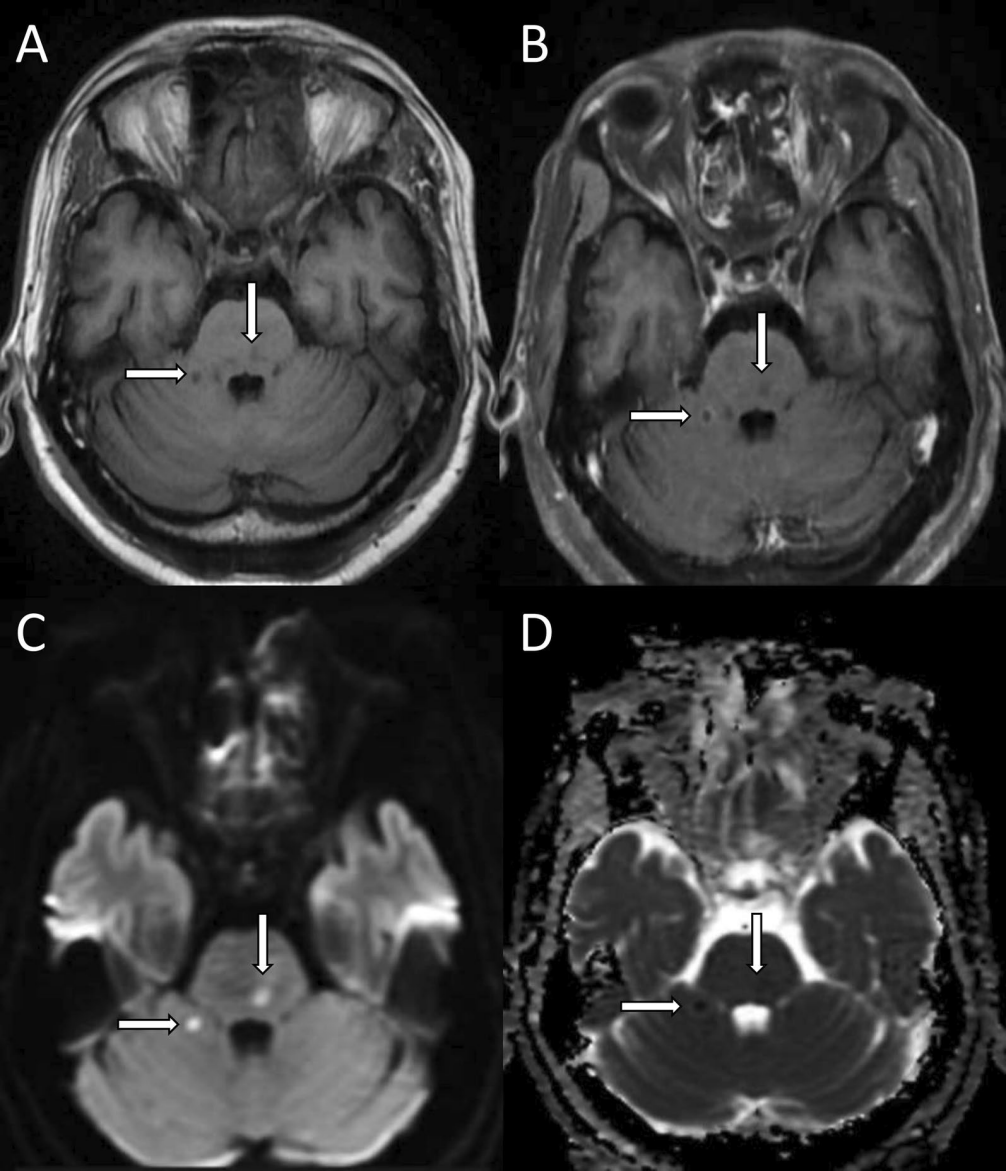
Septic emboli. Axial T1-weighted (**A**) and contrast-enhanced fat-saturated (**B**) images show lesions in the right pontocerebellar junction and pons with peripheral enhancement (white arrows). Diffusion-weighted and apparent diffusion coefficient (ADC) images (**C**, **D**) show foci of diffusion restriction (white arrows). In a known case of mucormycosis features indicate septic emboli

Infarcts and emboli both are consequences of vascular invasion of mucormycosis. Occlusion of the internal carotid artery can cause infarcts in the watershed territory better assessed with the help of DWI where they show restricted diffusion (Fig. 7). Basilar artery invasion, though uncommon in comparison with the internal carotid artery, can cause lesions in the posterior circulation [14, 22]. The infarcts due to vasculitis may be bland or laden with fungal hyphae [34]. Septic emboli appear as T1 hypointense, T2 hyperintense lesions with hyperintense edema on FLAIR and show peripheral enhancement and diffusion restriction on contrast-enhanced and diffusion-weighted magnetic resonance (MR) images, respectively (Fig. 8). They appear as hypointense microhemorrhages on SWI and are usually seen at the gray-white matter junction [35].

### Involvement of cranial nerves

Perineural spread, a well-recognized mode of spread in head and neck malignancies, is also seen in RCM. Nerve enlargement, irregularity, excessive post-contrast enhancement, and loss of fat space adjacent to the nerve at skull base foramen suggest nerve infiltration [20]. Diffusion restriction on DWI indicates nerve infarction. Fungal elements present in the intraconal compartment or orbital apex can invade the optic nerve sheath. Optic nerve infarction occurs on deep nerve infiltration or due to angioinvasion and occlusion of the central retinal or ophthalmic artery which leads to sudden irreversible blindness [36]. Involvement of cavernous sinus causes invasion of traversing nerves, which serves as a conduit for the spread of infection to the brainstem. Alternatively, fungal infection may ascend into the cranium through the involvement of nerves at the skull base and can lead to cavernous sinus thrombophlebitis/thrombosis. The trigeminal nerve and its branches serve as a major channel for the spread of infection along the floor of the cranial cavity [14, 20]. The olfactory nerves, with their projections into the basifrontal lobe and mesial temporal lobe, serve as important conduits for the preferential involvement of these regions [37].

### Skull base osteomyelitis

**Fig. 9:**
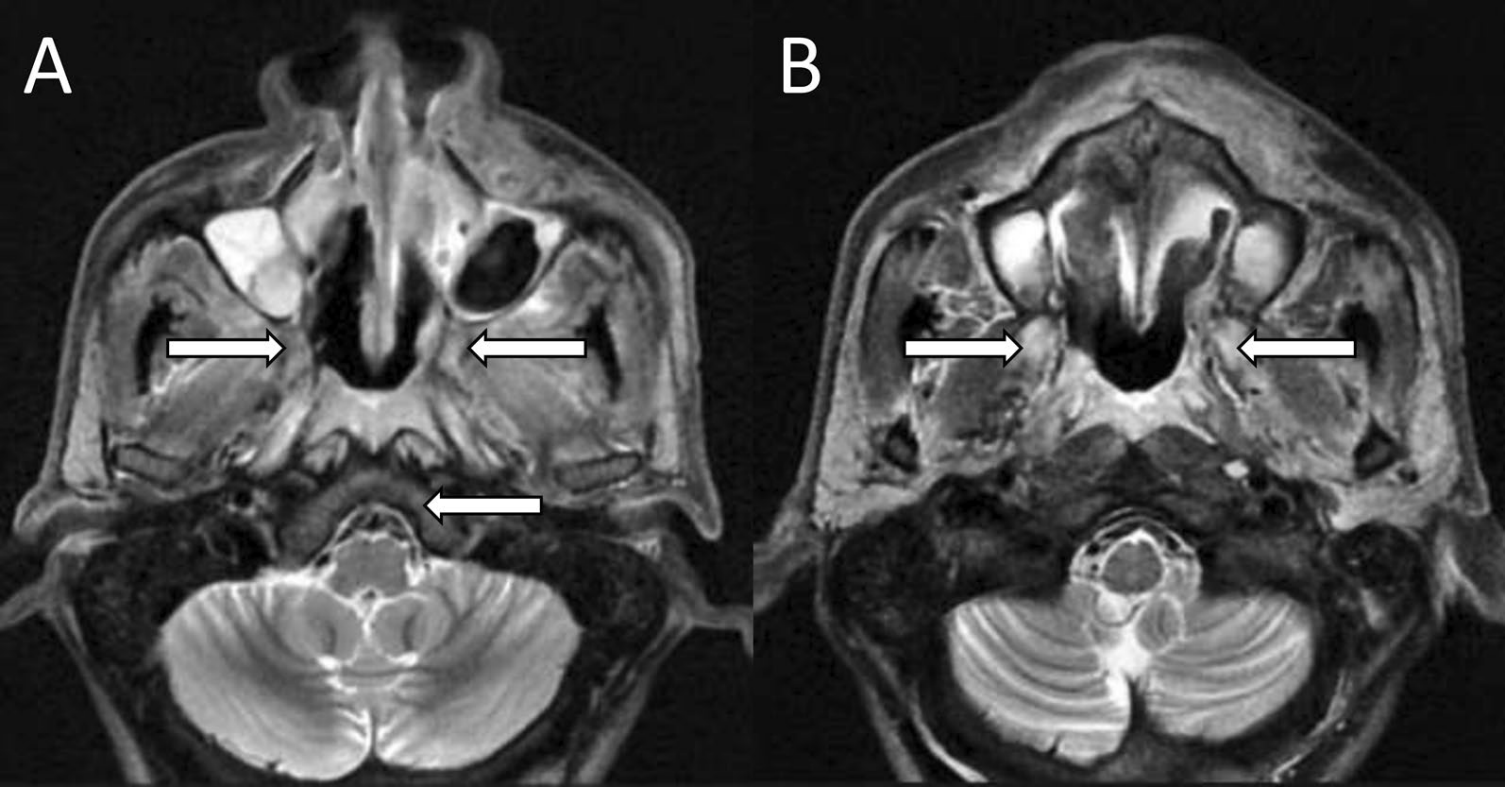
Skull base osteomyelitis. Axial STIR images (**A**, **B**) show patchy hyperintensity in the bilateral pterygoids and clivus (arrows), representing marrow edema suggestive of skull base involvement

Skull base involvement is rare and is seen in chronic cases. Bony infiltration seems inconvenient for the fungus when it can spread rapidly along the vascular channels. However, early bone involvement can appear as loss of normal fat signal on T1-weighted images, hyperintensity on STIR images, and heterogeneous enhancement on post-contrast MRI (Fig. 9) [38]. Late stages show rarefaction and erosion of bones, with large non-enhancing areas of devitalized tissue on contrast-enhanced images in and around the central skull base [39].

### Pachymeningitis

**Fig. 10:**
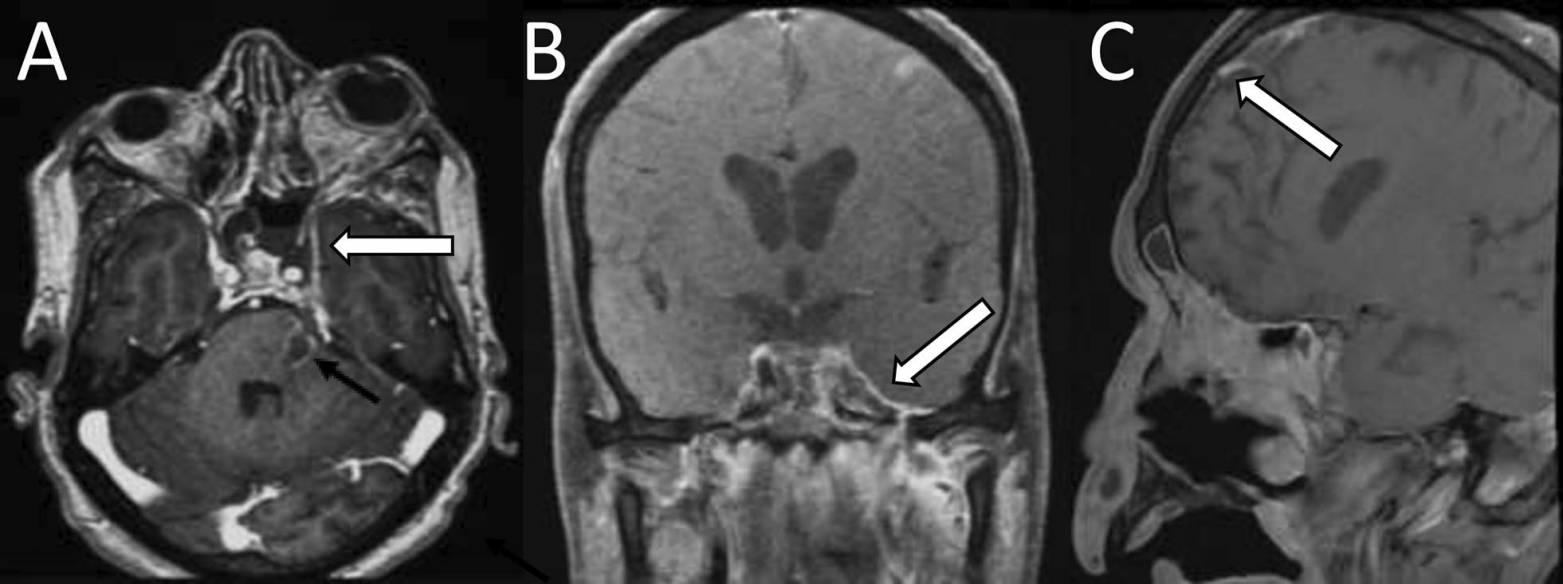
Pachymeningitis. Contrast-enhanced axial (**A**), coronal (**B**), and sagittal (**C**) T1-weighted fat-saturated images show pachymeningeal enhancement (white arrows) along the medial aspect of the left temporal and left frontal lobes. Image (**A**) also shows a ring-enhancing lesion in the pons suggesting a fungal abscess (black arrow)

Thickening of the dura with avid post-contrast enhancement is an early feature of intracranial involvement, although in some cases, it could be purely reactive (Fig. 10) [40]. It is usually seen along frontal convexities, in middle cranial fossa, and paracavernous region. It may be associated with ventriculitis and hydrocephalus.

### Aneurysm and hemorrhage

Mycotic aneurysm is a rare manifestation that is seen in the anterior circulation [30]. Rupture of aneurysm leads to subarachnoid hemorrhage which can be picked up well even on non-contrast CT scan. However, the detection of aneurysms requires angiographic studies.

## Conclusion

Intracranial extension of sinonasal mucormycosis is common, especially in diabetics, and is associated with high morbidity and mortality. Complications may range from meningitis to abscess formation and, rarely, subarachnoid hemorrhage. Due to its fulminant nature, a methodical approach should be followed meticulously while analyzing MR images. MRI is indispensable for rhinocerebral mucormycosis, and angiograms are necessary for a thorough assessment of vascular complications.

## Data Availability

The data are included in the manuscript.

## Abbreviations

CNS: Central nervous system
COVID-19: Coronavirus disease 2019
CT: Computed tomography
DWI: Diffusion-weighted imaging
FLAIR: Fluid-attenuated inverse recovery
FOV: Field of view
FSE: Fast spin echo
MR: Magnetic resonance
MRA: Magnetic resonance angiogram
MRI: Magnetic resonance imaging
MRS: Magnetic resonance spectroscopy
RCM: Rhinocerebral mucormycosis
SARS-CoV-2: Severe acute respiratory syndrome coronavirus 2
STIR: Short tau inverse recovery
SWI: Susceptibility-weighted imaging
TOF: Time of flight
